# Supplementary material for: Dnmt1 is essential for egg production and embryo viability in the large milkweed bug, Oncopeltus fasciatus
Source: Epigenetics Chromatin. 2019 Jan 7;12:6. doi: 10.1186/s13072-018-0246-5 (PMC6322253; doi:10.1186/s13072-018-0246-5)
Supplement: Supplementary file 1 — Additional file 1: Fig. S1. Identification of O. fasciatus DNA methyltransferases. a Phylogenetic relationship of DNA methyltransferases identified the de novo (Dnmt3) and maintenance (Dnmt1) DNA methyltransferase in O. fasciatus. Node support with ≤ 0.5 posterior probability is indicated—other nodes are ≥ 0.95. Branch lengths are in amino acid substitutions per site. Species names are represented as abbreviations: Acy. pis.: Acyrthosiphon pisum, Aed. aeg.: Aedes aegypti, Aed. alb.: Aedes albopictus, Ano. gam.: Anopheles gambiae, Api. mel.: A. mellifera, Bom. mor.: Bo. mori, Cam. flo.: Camponotus floridanus, Cop. flo.: Copidosoma floridanum, Cul. qui.: Culex pipiens quinquefasciatus, Dro. mel.: Drosophila melanogaster, Har. sal.: Harpegnathos saltator, Mic. dem.: Microplitis demolitor, Nas. vit.: Nasonia vitripennis, Nic. ves.: Nicrophorus vespilloides, Onc. fas.: O. fasciatus, Cer. bir.: Ooceraea (Cerapachys) biroi, Pol. can.: Polistes canadensis, Pol. dom.: Polistes dominula, Sol. inv.: Solenopsis invicta, Tri. cas.: Tribolium castaneum, and Zoo. nev.: Z. nevadensis. b A to scale representation of Dnmt1 and protein domains identified in O. fasciatus and M. musculus. Fig. S2. Assessment of RNAi treatment targeting S-adenosyl-L-methionine (AdoMet) region (ds-dnmt1-2) of Dnmt1 produces similar results as ds-dnmt1-1. a Assessment of RNAi treatment targeting Dnmt1 using qRT-PCR demonstrates successful reduction in transcription in ovaries compared to control. b Whole ovaries from ds-dnmt1-2 females removed 12–14 days post-injection. c Control follicular epithelium nuclei. d ds-dnmt1-1 follicular epithelium nuclei. e ds-dnmt1-2 follicular epithelium nuclei. For c–e scale bar corresponds to 100 µm. Fig. S3. DNA methylation consequences following posttranscriptional knockdown of Dnmt1 are restricted to ovaries. a Assessment of RNAi treatment targeting Dnmt1 using qRT-PCR demonstrates successful reduction in transcription compared to control across all tissues sampled. Col [file 13072_2018_246_MOESM1_ESM.zip › ESM/fig_s1.12052018.pdf]

Fig. S1

**a**

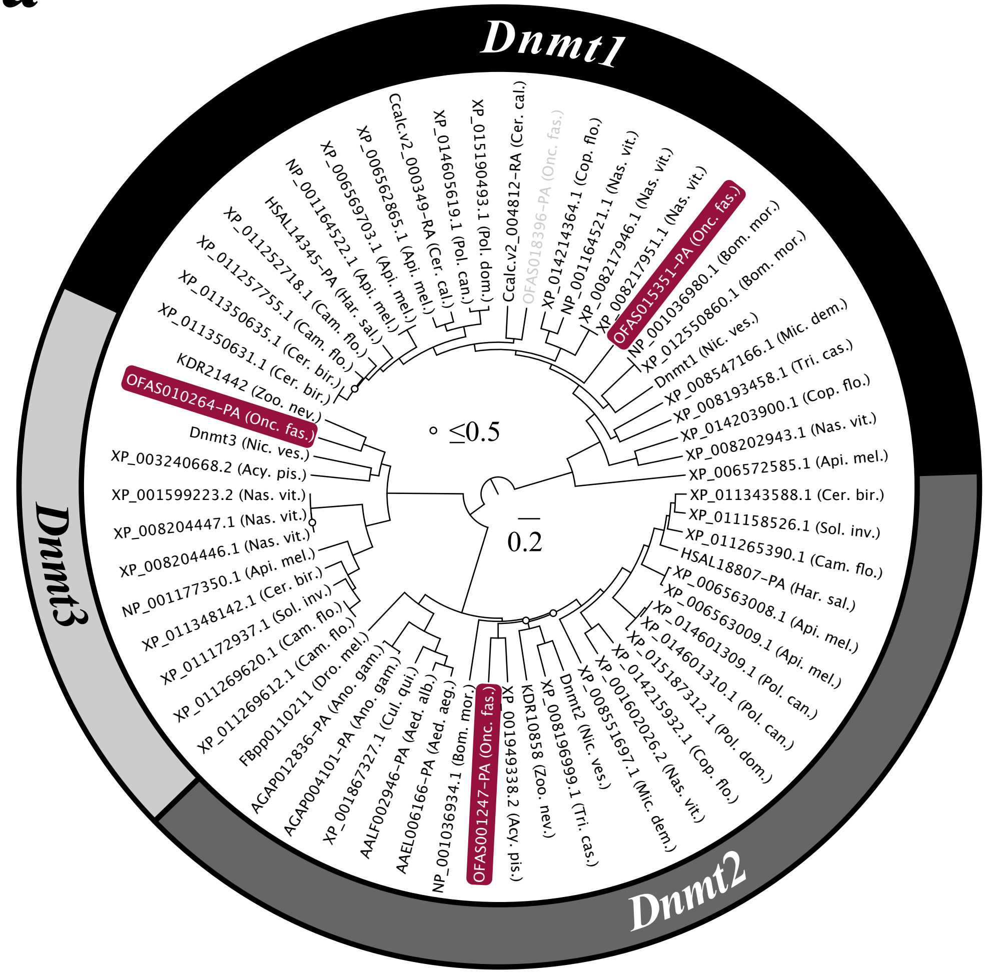
Dnmt1
Dnmt3
Dnmt2
≤0.5
0.2
OFAS015351-PA (Onc. fas.)
OFAS010264-PA (Onc. fas.)
OFAS001247-PA (Onc. fas.)
OFAS018396-PA (Onc. fas.)
XP_015190493.1 (Pol. dom.)
Ccalc.v2_004812-RA (Cer. cal.)
XP_014214364.1 (Cop. flo.)
NP_001164521.1 (Nas. vit.)
XP_008217946.1 (Nas. vit.)
XP_008217951.1 (Nas. vit.)
NP_001036980.1 (Bom. mor.)
XP_012550860.1 (Bom. mor.)
Dnmt1 (Nic. ves.)
XP_008547166.1 (Mic. dem.)
XP_008193458.1 (Tri. cas.)
XP_014203900.1 (Cop. flo.)
XP_008202943.1 (Nas. vit.)
XP_006572585.1 (Api. mel.)
XP_011343588.1 (Cer. bir.)
XP_011158526.1 (Sol. inv.)
XP_011265390.1 (Cam. flo.)
HSAL18807-PA (Har. sal.)
XP_006563008.1 (Api. mel.)
XP_006563009.1 (Api. mel.)
XP_014601309.1 (Pol. can.)
XP_014601310.1 (Pol. can.)
XP_015187312.1 (Pol. dom.)
XP_014215932.1 (Cop. flo.)
XP_001602026.2 (Nas. vit.)
XP_008551697.1 (Mic. dem.)
Dnmt2 (Nic. ves.)
XP_008196999.1 (Tri. cas.)
KDR10858 (Zoo. nev.)
XP_001949338.2 (Acy. pis.)
NP_001036934.1 (Bom. mor.)
AAEL006166-PA (Aed. aeg.)
AALF002946-PA (Aed. alb.)
XP_001867327.1 (Cul. qui.)
AGAP004101-PA (Ano. gam.)
ACAP012836-PA (Ano. gam.)
FBpp0110211 (Dro. mel.)
XP_011269612.1 (Cam. flo.)
XP_011269620.1 (Cam. flo.)
XP_011172937.1 (Sol. inv.)
XP_011348142.1 (Cer. bir.)
NP_001177350.1 (Api. mel.)
XP_008204446.1 (Nas. vit.)
XP_008204447.1 (Nas. vit.)
XP_001599223.2 (Nas. vit.)
XP_003240668.2 (Acy. pis.)
Dnmt3 (Nic. ves.)
KDR21442 (Zoo. nev.)
XP_011350631.1 (Cer. bir.)
XP_011350635.1 (Cer. bir.)
XP_011257755.1 (Cam. flo.)
XP_011252718.1 (Cam. flo.)
HSAL14345-PA (Har. sal.)
NP_001164522.1 (Api. mel.)
XP_006569703.1 (Api. mel.)
XP_006562865.1 (Api. mel.)
Ccalc.v2_000349-RA (Cer. cal.)
XP_014605619.1 (Pol. can.)

**b**

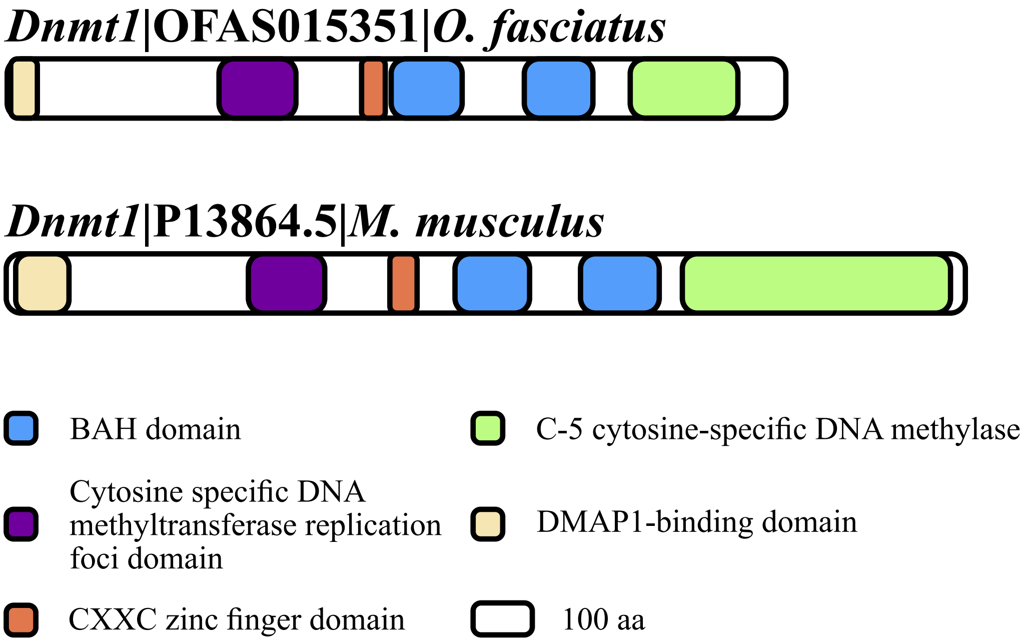
Dnmt1|OFAS015351|O. fasciatus
Dnmt1|P13864.5|M. musculus
BAH domain
C-5 cytosine-specific DNA methylase
Cytosine specific DNA methyltransferase replication foci domain
DMAP1-binding domain
CXXC zinc finger domain
100 aa
