# Supplementary figures and images for: Dnmt1 is essential for egg production and embryo viability in the large milkweed bug, Oncopeltus fasciatus
Source: Epigenetics Chromatin. 2019 Jan 7;12:6. doi: 10.1186/s13072-018-0246-5 (PMC6322253; doi:10.1186/s13072-018-0246-5)

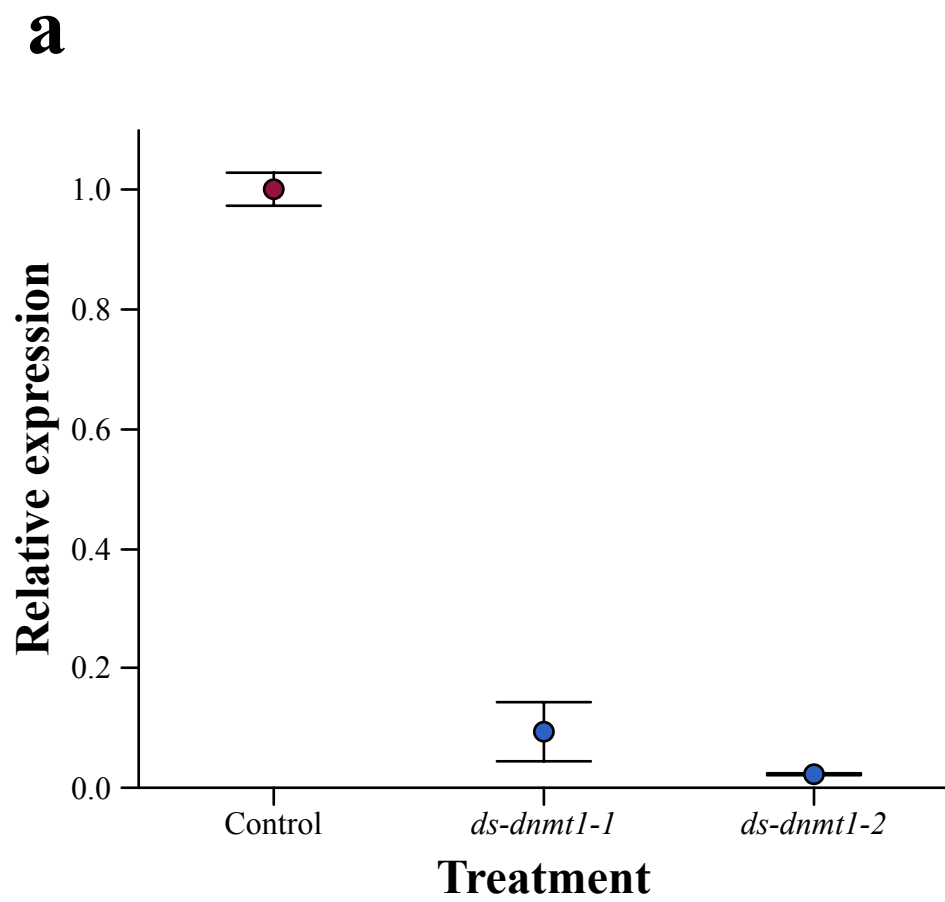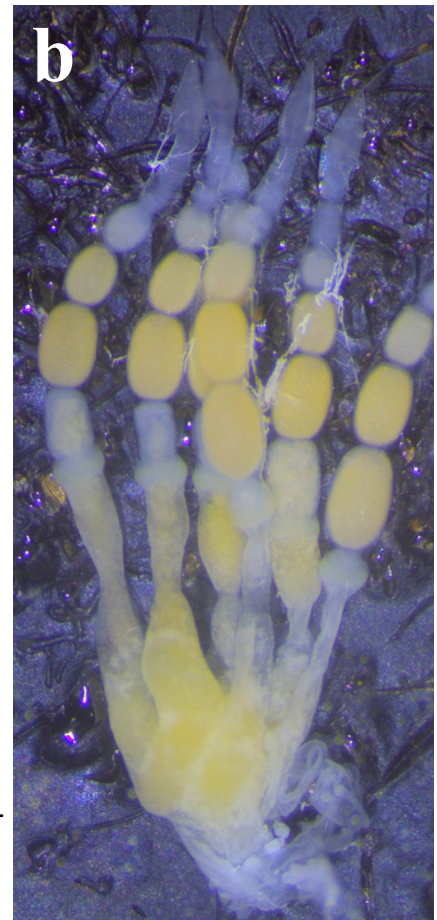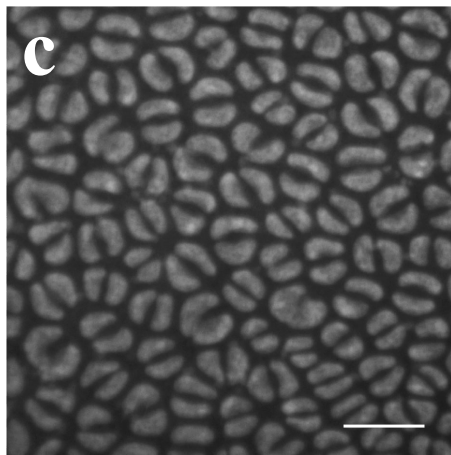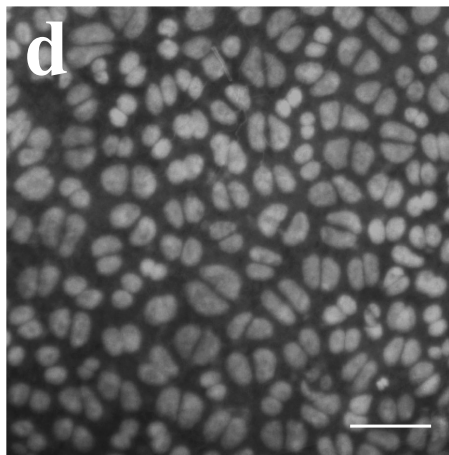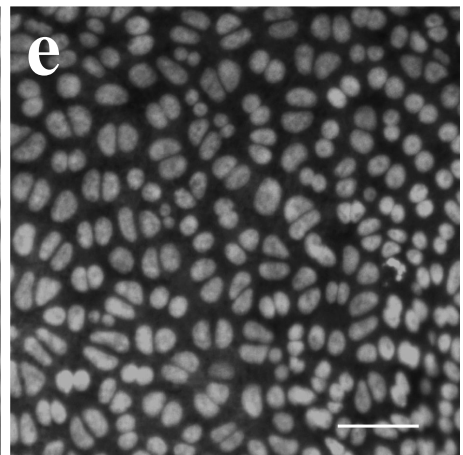

Supplement: Supplementary file 1 — Additional file 1: Fig. S1. Identification of O. fasciatus DNA methyltransferases. a Phylogenetic relationship of DNA methyltransferases identified the de novo (Dnmt3) and maintenance (Dnmt1) DNA methyltransferase in O. fasciatus. Node support with ≤ 0.5 posterior probability is indicated—other nodes are ≥ 0.95. Branch lengths are in amino acid substitutions per site. Species names are represented as abbreviations: Acy. pis.: Acyrthosiphon pisum, Aed. aeg.: Aedes aegypti, Aed. alb.: Aedes albopictus, Ano. gam.: Anopheles gambiae, Api. mel.: A. mellifera, Bom. mor.: Bo. mori, Cam. flo.: Camponotus floridanus, Cop. flo.: Copidosoma floridanum, Cul. qui.: Culex pipiens quinquefasciatus, Dro. mel.: Drosophila melanogaster, Har. sal.: Harpegnathos saltator, Mic. dem.: Microplitis demolitor, Nas. vit.: Nasonia vitripennis, Nic. ves.: Nicrophorus vespilloides, Onc. fas.: O. fasciatus, Cer. bir.: Ooceraea (Cerapachys) biroi, Pol. can.: Polistes canadensis, Pol. dom.: Polistes dominula, Sol. inv.: Solenopsis invicta, Tri. cas.: Tribolium castaneum, and Zoo. nev.: Z. nevadensis. b A to scale representation of Dnmt1 and protein domains identified in O. fasciatus and M. musculus. Fig. S2. Assessment of RNAi treatment targeting S-adenosyl-L-methionine (AdoMet) region (ds-dnmt1-2) of Dnmt1 produces similar results as ds-dnmt1-1. a Assessment of RNAi treatment targeting Dnmt1 using qRT-PCR demonstrates successful reduction in transcription in ovaries compared to control. b Whole ovaries from ds-dnmt1-2 females removed 12–14 days post-injection. c Control follicular epithelium nuclei. d ds-dnmt1-1 follicular epithelium nuclei. e ds-dnmt1-2 follicular epithelium nuclei. For c–e scale bar corresponds to 100 µm. Fig. S3. DNA methylation consequences following posttranscriptional knockdown of Dnmt1 are restricted to ovaries. a Assessment of RNAi treatment targeting Dnmt1 using qRT-PCR demonstrates successful reduction in transcription compared to control across all tissues sampled. Col [file 13072_2018_246_MOESM1_ESM.zip › ESM/fig_s2.12052018.pdf]

**a**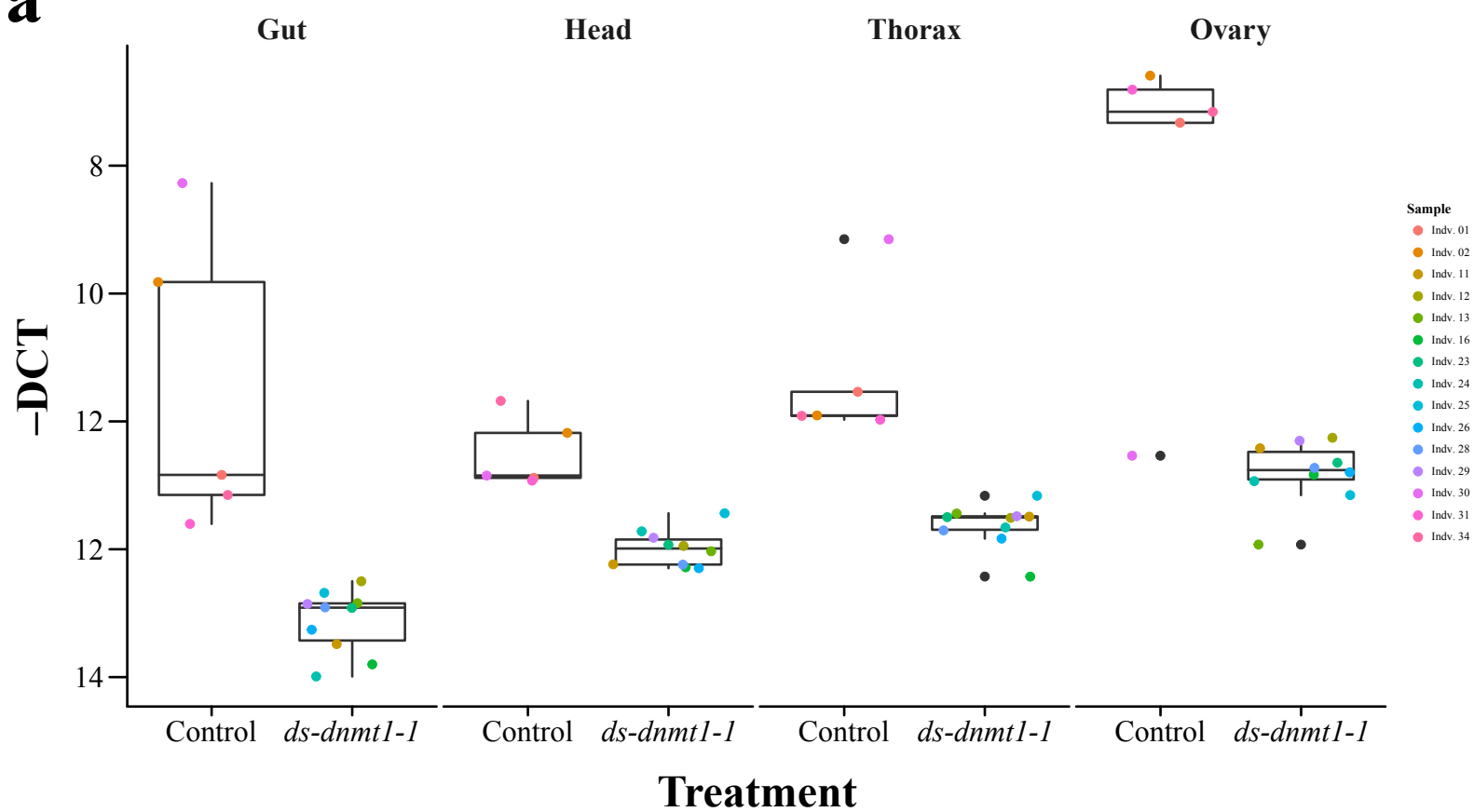**b**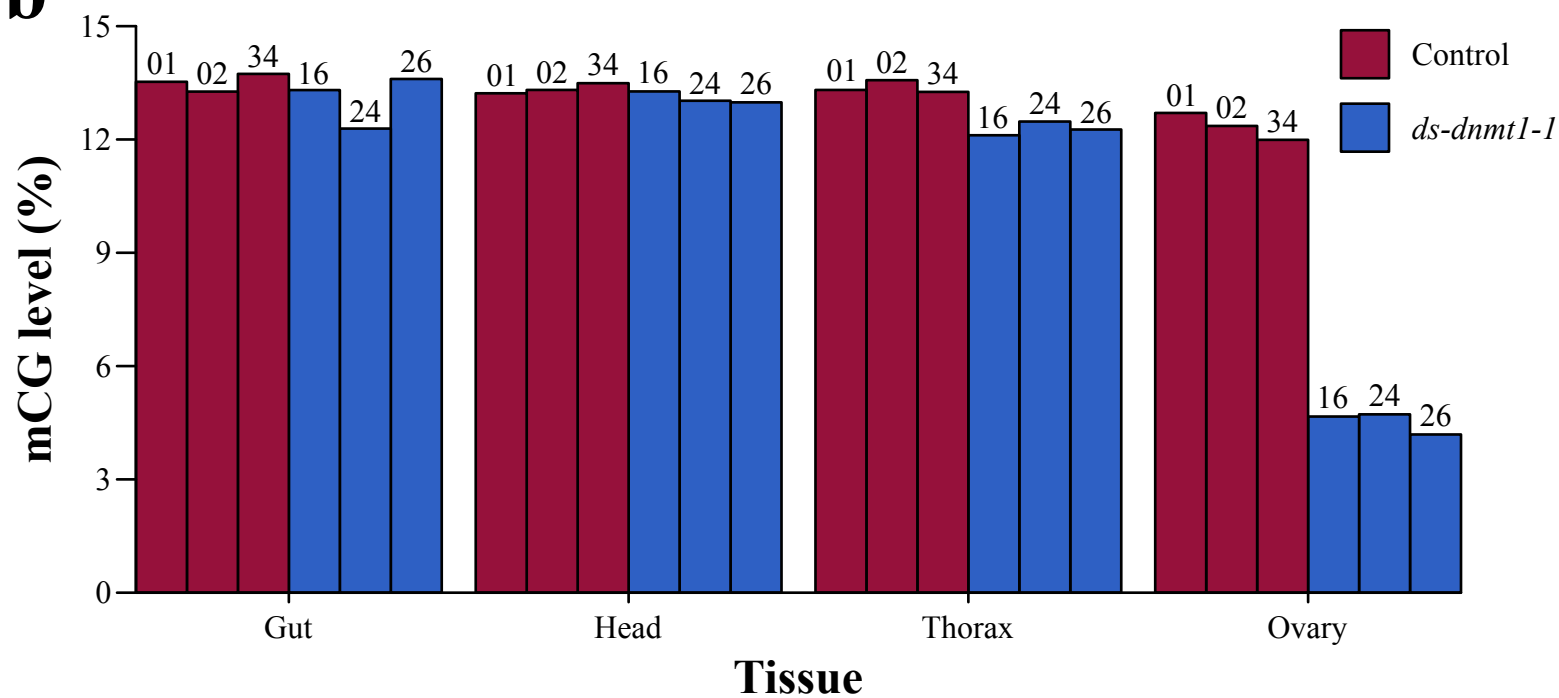

Supplement: Supplementary file 1 — Additional file 1: Fig. S1. Identification of O. fasciatus DNA methyltransferases. a Phylogenetic relationship of DNA methyltransferases identified the de novo (Dnmt3) and maintenance (Dnmt1) DNA methyltransferase in O. fasciatus. Node support with ≤ 0.5 posterior probability is indicated—other nodes are ≥ 0.95. Branch lengths are in amino acid substitutions per site. Species names are represented as abbreviations: Acy. pis.: Acyrthosiphon pisum, Aed. aeg.: Aedes aegypti, Aed. alb.: Aedes albopictus, Ano. gam.: Anopheles gambiae, Api. mel.: A. mellifera, Bom. mor.: Bo. mori, Cam. flo.: Camponotus floridanus, Cop. flo.: Copidosoma floridanum, Cul. qui.: Culex pipiens quinquefasciatus, Dro. mel.: Drosophila melanogaster, Har. sal.: Harpegnathos saltator, Mic. dem.: Microplitis demolitor, Nas. vit.: Nasonia vitripennis, Nic. ves.: Nicrophorus vespilloides, Onc. fas.: O. fasciatus, Cer. bir.: Ooceraea (Cerapachys) biroi, Pol. can.: Polistes canadensis, Pol. dom.: Polistes dominula, Sol. inv.: Solenopsis invicta, Tri. cas.: Tribolium castaneum, and Zoo. nev.: Z. nevadensis. b A to scale representation of Dnmt1 and protein domains identified in O. fasciatus and M. musculus. Fig. S2. Assessment of RNAi treatment targeting S-adenosyl-L-methionine (AdoMet) region (ds-dnmt1-2) of Dnmt1 produces similar results as ds-dnmt1-1. a Assessment of RNAi treatment targeting Dnmt1 using qRT-PCR demonstrates successful reduction in transcription in ovaries compared to control. b Whole ovaries from ds-dnmt1-2 females removed 12–14 days post-injection. c Control follicular epithelium nuclei. d ds-dnmt1-1 follicular epithelium nuclei. e ds-dnmt1-2 follicular epithelium nuclei. For c–e scale bar corresponds to 100 µm. Fig. S3. DNA methylation consequences following posttranscriptional knockdown of Dnmt1 are restricted to ovaries. a Assessment of RNAi treatment targeting Dnmt1 using qRT-PCR demonstrates successful reduction in transcription compared to control across all tissues sampled. Col [file 13072_2018_246_MOESM1_ESM.zip › ESM/fig_s3.12052018.pdf]

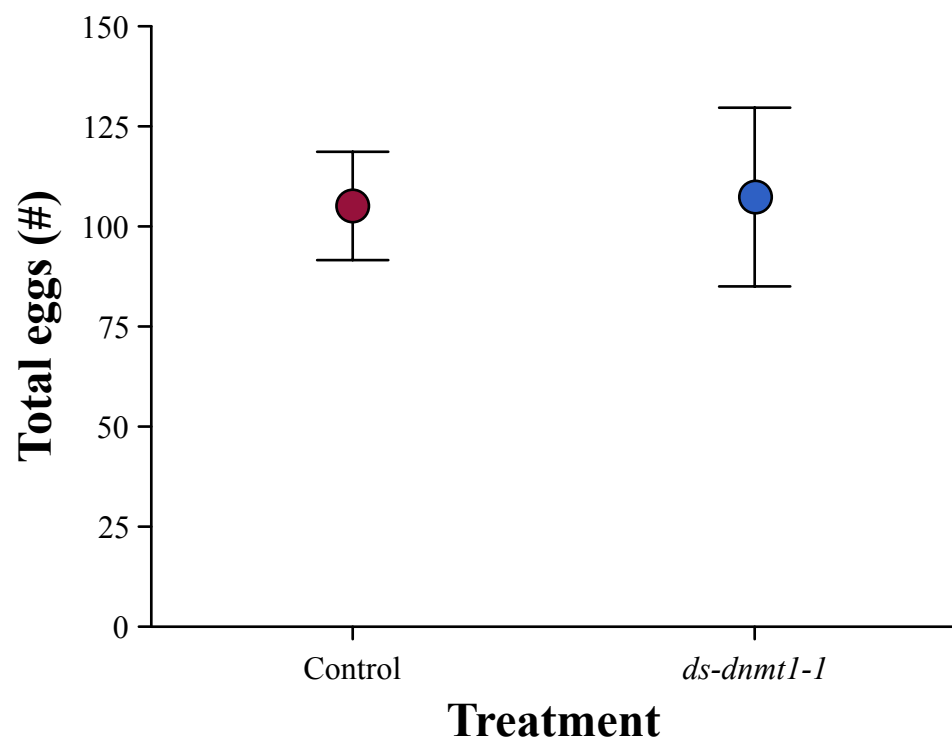

Supplement: Supplementary file 1 — Additional file 1: Fig. S1. Identification of O. fasciatus DNA methyltransferases. a Phylogenetic relationship of DNA methyltransferases identified the de novo (Dnmt3) and maintenance (Dnmt1) DNA methyltransferase in O. fasciatus. Node support with ≤ 0.5 posterior probability is indicated—other nodes are ≥ 0.95. Branch lengths are in amino acid substitutions per site. Species names are represented as abbreviations: Acy. pis.: Acyrthosiphon pisum, Aed. aeg.: Aedes aegypti, Aed. alb.: Aedes albopictus, Ano. gam.: Anopheles gambiae, Api. mel.: A. mellifera, Bom. mor.: Bo. mori, Cam. flo.: Camponotus floridanus, Cop. flo.: Copidosoma floridanum, Cul. qui.: Culex pipiens quinquefasciatus, Dro. mel.: Drosophila melanogaster, Har. sal.: Harpegnathos saltator, Mic. dem.: Microplitis demolitor, Nas. vit.: Nasonia vitripennis, Nic. ves.: Nicrophorus vespilloides, Onc. fas.: O. fasciatus, Cer. bir.: Ooceraea (Cerapachys) biroi, Pol. can.: Polistes canadensis, Pol. dom.: Polistes dominula, Sol. inv.: Solenopsis invicta, Tri. cas.: Tribolium castaneum, and Zoo. nev.: Z. nevadensis. b A to scale representation of Dnmt1 and protein domains identified in O. fasciatus and M. musculus. Fig. S2. Assessment of RNAi treatment targeting S-adenosyl-L-methionine (AdoMet) region (ds-dnmt1-2) of Dnmt1 produces similar results as ds-dnmt1-1. a Assessment of RNAi treatment targeting Dnmt1 using qRT-PCR demonstrates successful reduction in transcription in ovaries compared to control. b Whole ovaries from ds-dnmt1-2 females removed 12–14 days post-injection. c Control follicular epithelium nuclei. d ds-dnmt1-1 follicular epithelium nuclei. e ds-dnmt1-2 follicular epithelium nuclei. For c–e scale bar corresponds to 100 µm. Fig. S3. DNA methylation consequences following posttranscriptional knockdown of Dnmt1 are restricted to ovaries. a Assessment of RNAi treatment targeting Dnmt1 using qRT-PCR demonstrates successful reduction in transcription compared to control across all tissues sampled. Col [file 13072_2018_246_MOESM1_ESM.zip › ESM/fig_s4.12052018.pdf]

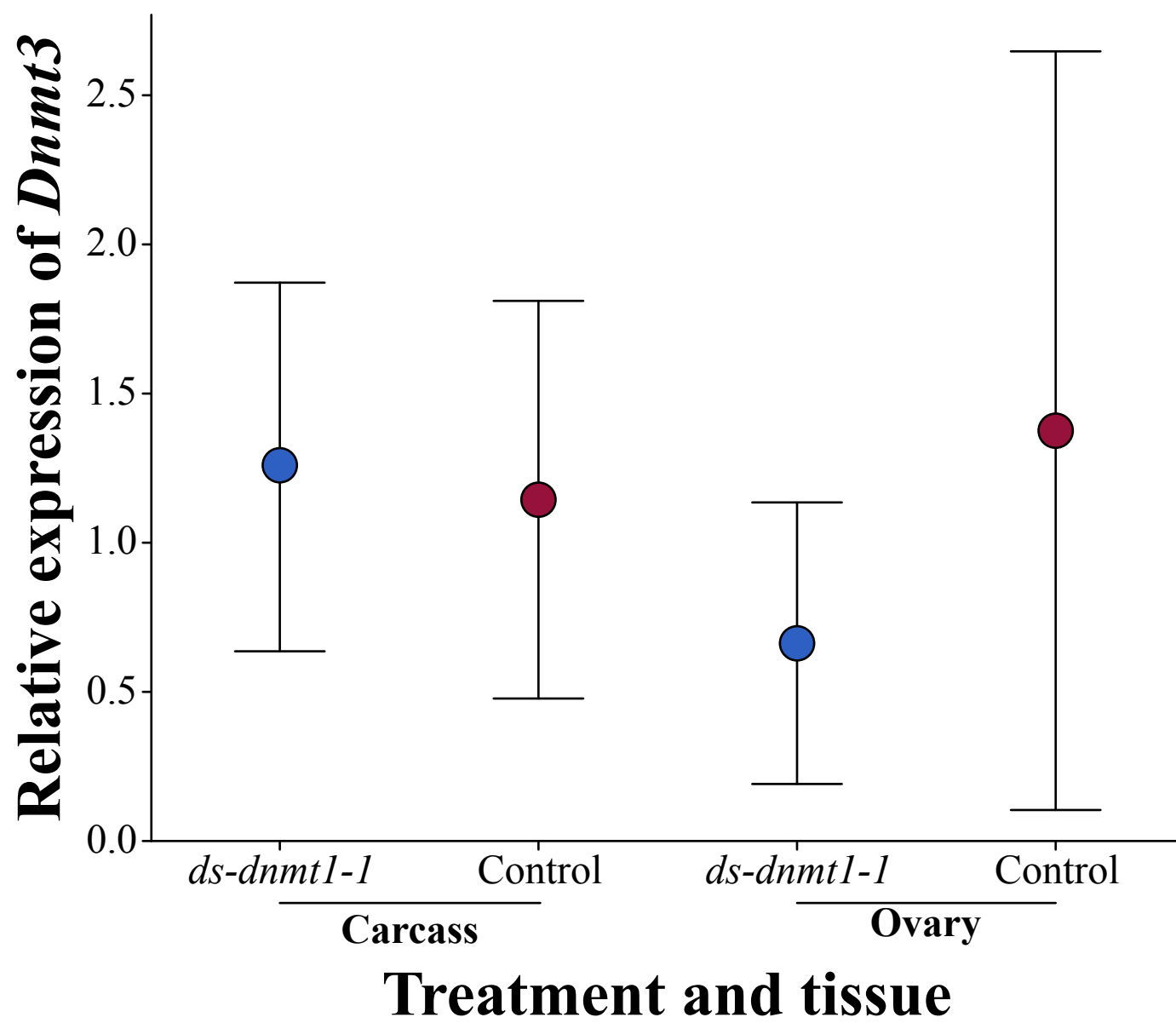

Supplement: Supplementary file 1 — Additional file 1: Fig. S1. Identification of O. fasciatus DNA methyltransferases. a Phylogenetic relationship of DNA methyltransferases identified the de novo (Dnmt3) and maintenance (Dnmt1) DNA methyltransferase in O. fasciatus. Node support with ≤ 0.5 posterior probability is indicated—other nodes are ≥ 0.95. Branch lengths are in amino acid substitutions per site. Species names are represented as abbreviations: Acy. pis.: Acyrthosiphon pisum, Aed. aeg.: Aedes aegypti, Aed. alb.: Aedes albopictus, Ano. gam.: Anopheles gambiae, Api. mel.: A. mellifera, Bom. mor.: Bo. mori, Cam. flo.: Camponotus floridanus, Cop. flo.: Copidosoma floridanum, Cul. qui.: Culex pipiens quinquefasciatus, Dro. mel.: Drosophila melanogaster, Har. sal.: Harpegnathos saltator, Mic. dem.: Microplitis demolitor, Nas. vit.: Nasonia vitripennis, Nic. ves.: Nicrophorus vespilloides, Onc. fas.: O. fasciatus, Cer. bir.: Ooceraea (Cerapachys) biroi, Pol. can.: Polistes canadensis, Pol. dom.: Polistes dominula, Sol. inv.: Solenopsis invicta, Tri. cas.: Tribolium castaneum, and Zoo. nev.: Z. nevadensis. b A to scale representation of Dnmt1 and protein domains identified in O. fasciatus and M. musculus. Fig. S2. Assessment of RNAi treatment targeting S-adenosyl-L-methionine (AdoMet) region (ds-dnmt1-2) of Dnmt1 produces similar results as ds-dnmt1-1. a Assessment of RNAi treatment targeting Dnmt1 using qRT-PCR demonstrates successful reduction in transcription in ovaries compared to control. b Whole ovaries from ds-dnmt1-2 females removed 12–14 days post-injection. c Control follicular epithelium nuclei. d ds-dnmt1-1 follicular epithelium nuclei. e ds-dnmt1-2 follicular epithelium nuclei. For c–e scale bar corresponds to 100 µm. Fig. S3. DNA methylation consequences following posttranscriptional knockdown of Dnmt1 are restricted to ovaries. a Assessment of RNAi treatment targeting Dnmt1 using qRT-PCR demonstrates successful reduction in transcription compared to control across all tissues sampled. Col [file 13072_2018_246_MOESM1_ESM.zip › ESM/fig_s5.12052018.pdf]

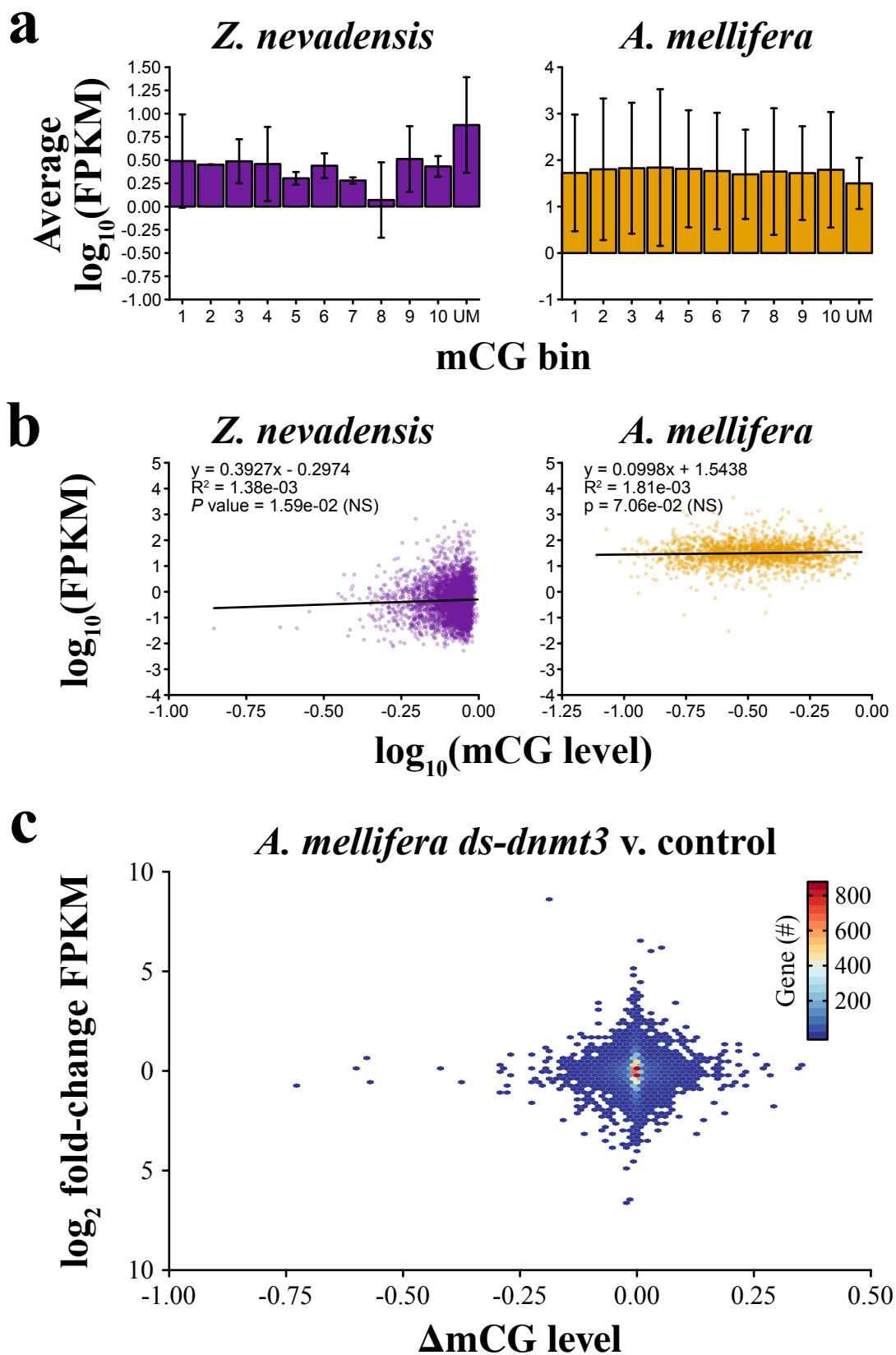

Supplement: Supplementary file 1 — Additional file 1: Fig. S1. Identification of O. fasciatus DNA methyltransferases. a Phylogenetic relationship of DNA methyltransferases identified the de novo (Dnmt3) and maintenance (Dnmt1) DNA methyltransferase in O. fasciatus. Node support with ≤ 0.5 posterior probability is indicated—other nodes are ≥ 0.95. Branch lengths are in amino acid substitutions per site. Species names are represented as abbreviations: Acy. pis.: Acyrthosiphon pisum, Aed. aeg.: Aedes aegypti, Aed. alb.: Aedes albopictus, Ano. gam.: Anopheles gambiae, Api. mel.: A. mellifera, Bom. mor.: Bo. mori, Cam. flo.: Camponotus floridanus, Cop. flo.: Copidosoma floridanum, Cul. qui.: Culex pipiens quinquefasciatus, Dro. mel.: Drosophila melanogaster, Har. sal.: Harpegnathos saltator, Mic. dem.: Microplitis demolitor, Nas. vit.: Nasonia vitripennis, Nic. ves.: Nicrophorus vespilloides, Onc. fas.: O. fasciatus, Cer. bir.: Ooceraea (Cerapachys) biroi, Pol. can.: Polistes canadensis, Pol. dom.: Polistes dominula, Sol. inv.: Solenopsis invicta, Tri. cas.: Tribolium castaneum, and Zoo. nev.: Z. nevadensis. b A to scale representation of Dnmt1 and protein domains identified in O. fasciatus and M. musculus. Fig. S2. Assessment of RNAi treatment targeting S-adenosyl-L-methionine (AdoMet) region (ds-dnmt1-2) of Dnmt1 produces similar results as ds-dnmt1-1. a Assessment of RNAi treatment targeting Dnmt1 using qRT-PCR demonstrates successful reduction in transcription in ovaries compared to control. b Whole ovaries from ds-dnmt1-2 females removed 12–14 days post-injection. c Control follicular epithelium nuclei. d ds-dnmt1-1 follicular epithelium nuclei. e ds-dnmt1-2 follicular epithelium nuclei. For c–e scale bar corresponds to 100 µm. Fig. S3. DNA methylation consequences following posttranscriptional knockdown of Dnmt1 are restricted to ovaries. a Assessment of RNAi treatment targeting Dnmt1 using qRT-PCR demonstrates successful reduction in transcription compared to control across all tissues sampled. Col [file 13072_2018_246_MOESM1_ESM.zip › ESM/fig_s6.12052018.pdf]

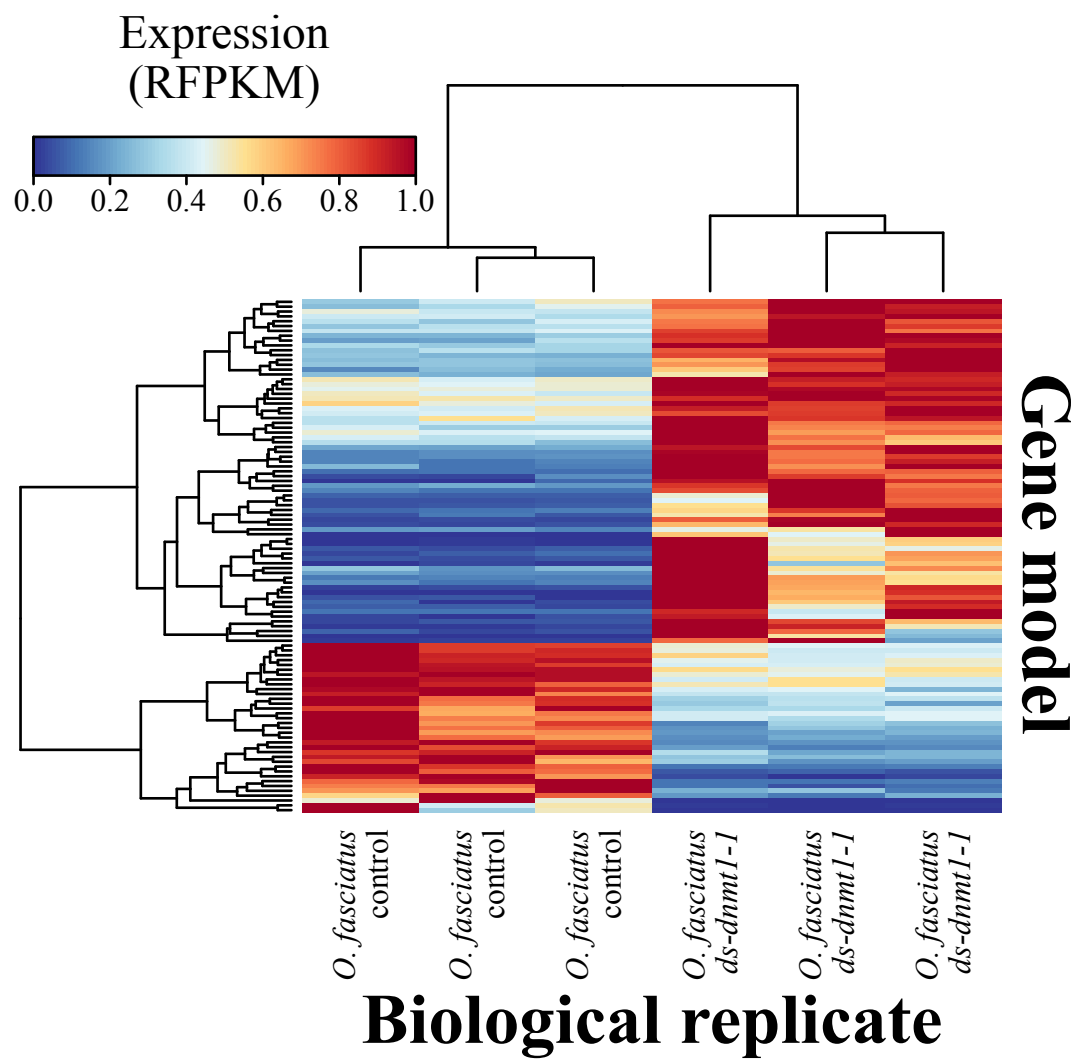

Supplement: Supplementary file 1 — Additional file 1: Fig. S1. Identification of O. fasciatus DNA methyltransferases. a Phylogenetic relationship of DNA methyltransferases identified the de novo (Dnmt3) and maintenance (Dnmt1) DNA methyltransferase in O. fasciatus. Node support with ≤ 0.5 posterior probability is indicated—other nodes are ≥ 0.95. Branch lengths are in amino acid substitutions per site. Species names are represented as abbreviations: Acy. pis.: Acyrthosiphon pisum, Aed. aeg.: Aedes aegypti, Aed. alb.: Aedes albopictus, Ano. gam.: Anopheles gambiae, Api. mel.: A. mellifera, Bom. mor.: Bo. mori, Cam. flo.: Camponotus floridanus, Cop. flo.: Copidosoma floridanum, Cul. qui.: Culex pipiens quinquefasciatus, Dro. mel.: Drosophila melanogaster, Har. sal.: Harpegnathos saltator, Mic. dem.: Microplitis demolitor, Nas. vit.: Nasonia vitripennis, Nic. ves.: Nicrophorus vespilloides, Onc. fas.: O. fasciatus, Cer. bir.: Ooceraea (Cerapachys) biroi, Pol. can.: Polistes canadensis, Pol. dom.: Polistes dominula, Sol. inv.: Solenopsis invicta, Tri. cas.: Tribolium castaneum, and Zoo. nev.: Z. nevadensis. b A to scale representation of Dnmt1 and protein domains identified in O. fasciatus and M. musculus. Fig. S2. Assessment of RNAi treatment targeting S-adenosyl-L-methionine (AdoMet) region (ds-dnmt1-2) of Dnmt1 produces similar results as ds-dnmt1-1. a Assessment of RNAi treatment targeting Dnmt1 using qRT-PCR demonstrates successful reduction in transcription in ovaries compared to control. b Whole ovaries from ds-dnmt1-2 females removed 12–14 days post-injection. c Control follicular epithelium nuclei. d ds-dnmt1-1 follicular epithelium nuclei. e ds-dnmt1-2 follicular epithelium nuclei. For c–e scale bar corresponds to 100 µm. Fig. S3. DNA methylation consequences following posttranscriptional knockdown of Dnmt1 are restricted to ovaries. a Assessment of RNAi treatment targeting Dnmt1 using qRT-PCR demonstrates successful reduction in transcription compared to control across all tissues sampled. Col [file 13072_2018_246_MOESM1_ESM.zip › ESM/fig_s7.12052018.pdf]

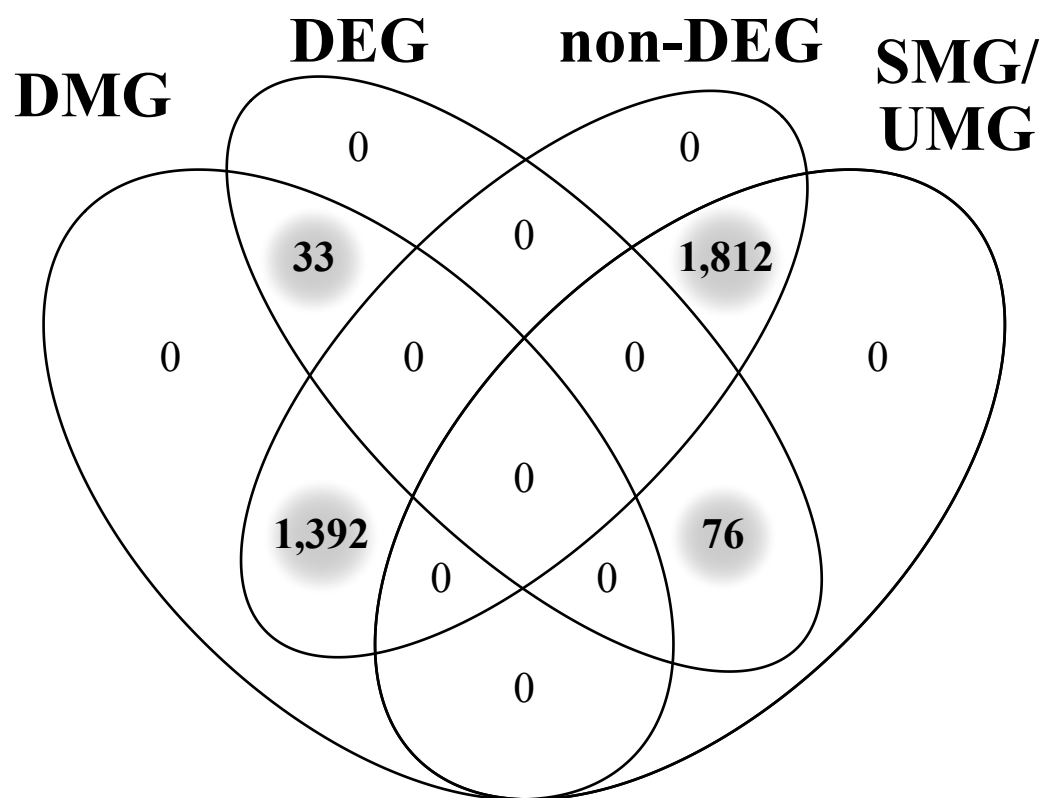

Supplement: Supplementary file 1 — Additional file 1: Fig. S1. Identification of O. fasciatus DNA methyltransferases. a Phylogenetic relationship of DNA methyltransferases identified the de novo (Dnmt3) and maintenance (Dnmt1) DNA methyltransferase in O. fasciatus. Node support with ≤ 0.5 posterior probability is indicated—other nodes are ≥ 0.95. Branch lengths are in amino acid substitutions per site. Species names are represented as abbreviations: Acy. pis.: Acyrthosiphon pisum, Aed. aeg.: Aedes aegypti, Aed. alb.: Aedes albopictus, Ano. gam.: Anopheles gambiae, Api. mel.: A. mellifera, Bom. mor.: Bo. mori, Cam. flo.: Camponotus floridanus, Cop. flo.: Copidosoma floridanum, Cul. qui.: Culex pipiens quinquefasciatus, Dro. mel.: Drosophila melanogaster, Har. sal.: Harpegnathos saltator, Mic. dem.: Microplitis demolitor, Nas. vit.: Nasonia vitripennis, Nic. ves.: Nicrophorus vespilloides, Onc. fas.: O. fasciatus, Cer. bir.: Ooceraea (Cerapachys) biroi, Pol. can.: Polistes canadensis, Pol. dom.: Polistes dominula, Sol. inv.: Solenopsis invicta, Tri. cas.: Tribolium castaneum, and Zoo. nev.: Z. nevadensis. b A to scale representation of Dnmt1 and protein domains identified in O. fasciatus and M. musculus. Fig. S2. Assessment of RNAi treatment targeting S-adenosyl-L-methionine (AdoMet) region (ds-dnmt1-2) of Dnmt1 produces similar results as ds-dnmt1-1. a Assessment of RNAi treatment targeting Dnmt1 using qRT-PCR demonstrates successful reduction in transcription in ovaries compared to control. b Whole ovaries from ds-dnmt1-2 females removed 12–14 days post-injection. c Control follicular epithelium nuclei. d ds-dnmt1-1 follicular epithelium nuclei. e ds-dnmt1-2 follicular epithelium nuclei. For c–e scale bar corresponds to 100 µm. Fig. S3. DNA methylation consequences following posttranscriptional knockdown of Dnmt1 are restricted to ovaries. a Assessment of RNAi treatment targeting Dnmt1 using qRT-PCR demonstrates successful reduction in transcription compared to control across all tissues sampled. Col [file 13072_2018_246_MOESM1_ESM.zip › ESM/fig_s8.12052018.pdf]

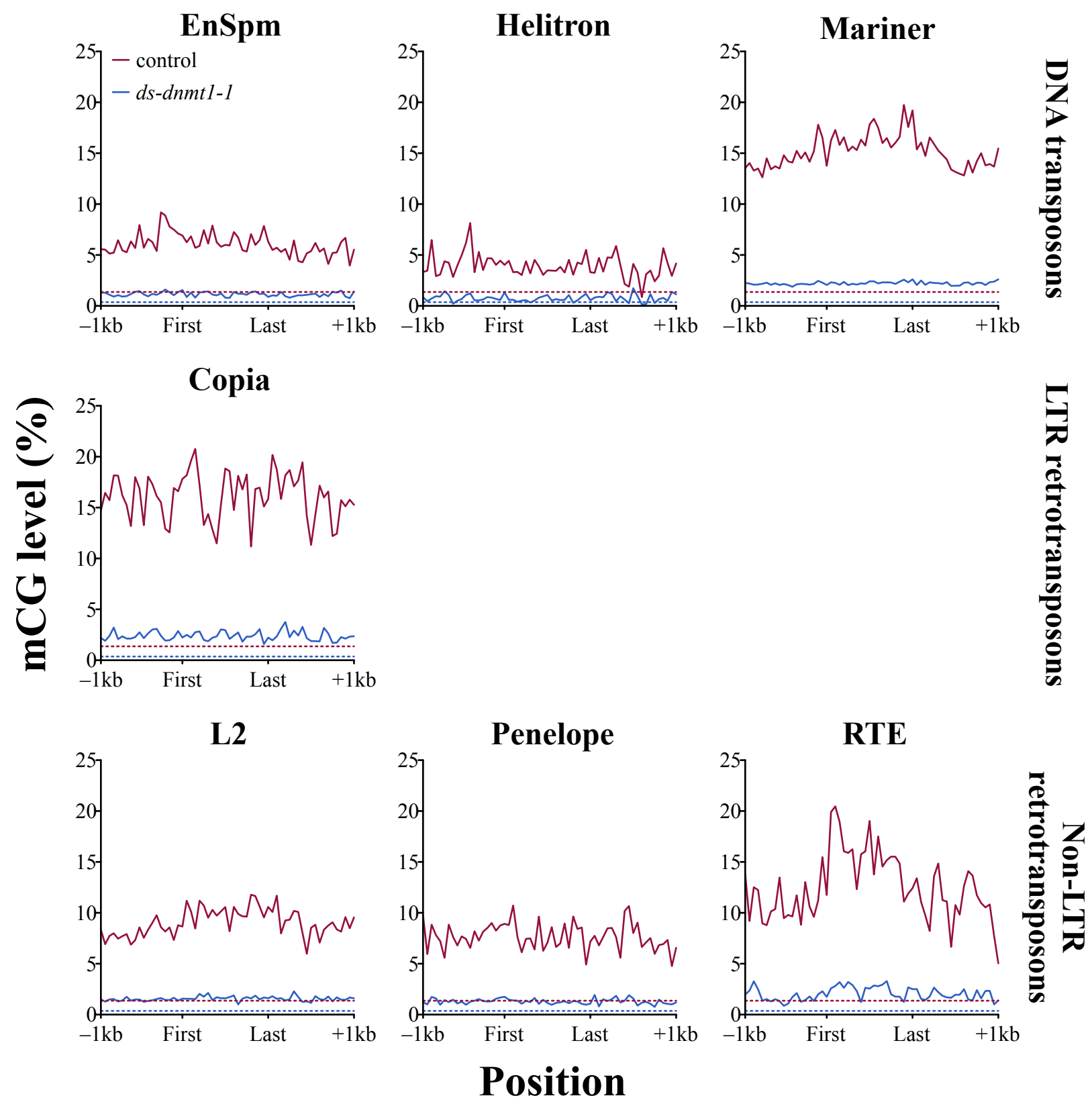

Supplement: Supplementary file 1 — Additional file 1: Fig. S1. Identification of O. fasciatus DNA methyltransferases. a Phylogenetic relationship of DNA methyltransferases identified the de novo (Dnmt3) and maintenance (Dnmt1) DNA methyltransferase in O. fasciatus. Node support with ≤ 0.5 posterior probability is indicated—other nodes are ≥ 0.95. Branch lengths are in amino acid substitutions per site. Species names are represented as abbreviations: Acy. pis.: Acyrthosiphon pisum, Aed. aeg.: Aedes aegypti, Aed. alb.: Aedes albopictus, Ano. gam.: Anopheles gambiae, Api. mel.: A. mellifera, Bom. mor.: Bo. mori, Cam. flo.: Camponotus floridanus, Cop. flo.: Copidosoma floridanum, Cul. qui.: Culex pipiens quinquefasciatus, Dro. mel.: Drosophila melanogaster, Har. sal.: Harpegnathos saltator, Mic. dem.: Microplitis demolitor, Nas. vit.: Nasonia vitripennis, Nic. ves.: Nicrophorus vespilloides, Onc. fas.: O. fasciatus, Cer. bir.: Ooceraea (Cerapachys) biroi, Pol. can.: Polistes canadensis, Pol. dom.: Polistes dominula, Sol. inv.: Solenopsis invicta, Tri. cas.: Tribolium castaneum, and Zoo. nev.: Z. nevadensis. b A to scale representation of Dnmt1 and protein domains identified in O. fasciatus and M. musculus. Fig. S2. Assessment of RNAi treatment targeting S-adenosyl-L-methionine (AdoMet) region (ds-dnmt1-2) of Dnmt1 produces similar results as ds-dnmt1-1. a Assessment of RNAi treatment targeting Dnmt1 using qRT-PCR demonstrates successful reduction in transcription in ovaries compared to control. b Whole ovaries from ds-dnmt1-2 females removed 12–14 days post-injection. c Control follicular epithelium nuclei. d ds-dnmt1-1 follicular epithelium nuclei. e ds-dnmt1-2 follicular epithelium nuclei. For c–e scale bar corresponds to 100 µm. Fig. S3. DNA methylation consequences following posttranscriptional knockdown of Dnmt1 are restricted to ovaries. a Assessment of RNAi treatment targeting Dnmt1 using qRT-PCR demonstrates successful reduction in transcription compared to control across all tissues sampled. Col [file 13072_2018_246_MOESM1_ESM.zip › ESM/fig_s9.12052018.pdf]
